# Development of tools to automate quantitative analysis of radiation damage in SAXS experiments

**DOI:** 10.1107/S1600577516015083

**Published:** 2017-01-01

**Authors:** Jonathan C. Brooks-Bartlett, Rebecca A. Batters, Charles S. Bury, Edward D. Lowe, Helen Mary Ginn, Adam Round, Elspeth F. Garman

**Affiliations:** a Department of Biochemistry, University of Oxford, Oxford OX1 3QU, UK; b Division of Structural Biology, Wellcome Trust Centre for Human Genetics, Roosevelt Drive, Oxford OX3 7BN, UK; c European Molecular Biology Laboratory, Grenoble Outstation, 71 avenue des Martyrs, CS 90181, 38042 Grenoble, France; d SPB/SFX European XFEL, Holzkoppel 4, 22869 Schenefeld, Germany; e Faculty of Natural Sciences, Keele University, Staffordshire ST5 5BG, UK

**Keywords:** SAXS, radiation damage, *RADDOSE-3D*, radioprotectants, CorMap visualization

## Abstract

Biological small-angle X-ray scattering (SAXS) is an increasingly popular technique used to obtain nanoscale structural information on macromolecules in solution. However, radiation damage to the samples limits the amount of useful data that can be collected from a single sample. In contrast to the extensive analytical resources available for macromolecular crystallography (MX), there are relatively few tools to quantitate radiation damage for SAXS, some of which require a significant level of manual characterization, with the potential of leading to conflicting results from different studies. Here, computational tools have been developed to automate and standardize radiation damage analysis for SAXS data. *RADDOSE-3D*, a dose calculation software utility originally written for MX experiments, has been extended to account for the cylindrical geometry of the capillary tube, the liquid composition of the sample and the attenuation of the beam by the capillary material to allow doses to be calculated for many SAXS experiments. Furthermore, a library has been written to visualize and explore the pairwise similarity of frames. The calculated dose for the frame at which three subsequent frames are determined to be dissimilar is defined as the radiation damage onset threshold (RDOT). Analysis of RDOTs has been used to compare the efficacy of radioprotectant compounds to extend the useful lifetime of SAXS samples. Comparison of the RDOTs shows that, for radioprotectant compounds at 5 and 10 m*M* concentration, glycerol is the most effective compound. However, at 1 and 2 m*M* concentrations, di­thio­threitol (DTT) appears to be most effective. Our newly developed visualization library contains methods that highlight the unusual radiation damage results given by SAXS data collected using higher concentrations of DTT: these observations should pave the way to the development of more sophisticated frame merging strategies.

## Abstract 2

Radiation damage analysis with experimental SAXS data allows for the quantitative comparison of the efficacy of various additive radioprotectant compounds. Relevant extensions to *RADDOSE-3D* and the creation of a new visualization library to enable this study are presented.

## 1. Introduction   

Biological small-angle X-ray scattering (SAXS) is an experimental technique that provides low-resolution structural information on macromolecules. The surge in popularity of the technique is a result of recent improvements in both software and hardware, allowing for high-throughput data collection and analysis (Bizien *et al.*, 2016 ▸; Graewert & Svergun, 2013 ▸). This is reflected in the increasing number of dedicated SAXS beamlines such as BM29 at the ESRF, P12 at the EMBL Hamburg and B21 at Diamond Light Source (Blanchet *et al.*, 2015 ▸; Brennich *et al.*, 2016 ▸; Materlik *et al.*, 2015 ▸; Pernot *et al.*, 2013 ▸).

However, as for most other macromolecular structural techniques, radiation damage is still a major factor hindering the success of experiments. The high solvent proportion of biological SAXS samples means that hydroxyl, hydro­peroxyl radicals and hydrated electrons are produced in abundance by the radiolysis of water when it is irradiated with X-rays (Allan *et al.*, 2013 ▸; Garman, 2010 ▸; Kuwamoto *et al.*, 2004 ▸). These radicals can then interact with the protein molecules, ultimately leading to protein aggregation, fragmentation or unfolding (Garrison, 1987 ▸; Hopkins & Thorne, 2016 ▸; Houée-Lévin *et al.*, 2015 ▸; Kuwamoto *et al.*, 2004 ▸). The categories of these radiation-induced processes can generally be distinguished by their various effects on the SAXS data. Aggregation manifests as an increase in scattering intensity at low angles, whereas fragmentation has the opposite effect, resulting in a decrease in scattering at low angles (Jeffries *et al.*, 2015 ▸; Kuwamoto *et al.*, 2004 ▸). Furthermore, molecular repulsion due to protein charging can also decrease the scattering at low angles. Unfolding, on the other hand, results in an increase in the radius of gyration and a decrease in the Porod exponent (Hopkins & Thorne, 2016 ▸).

Common methods used to reduce radiation damage to biological SAXS samples are generally concerned with limiting the X-ray exposure to any given volume of sample. These include: flowing or oscillating the sample in the container (usually a quartz or glass capillary, but could also be a flat cell with mica windows or a printed solution holder), reducing the exposure time, and attenuation or defocusing of the X-ray beam (Fischetti *et al.*, 2003 ▸; Jeffries *et al.*, 2015 ▸; Martel *et al.*, 2012 ▸; Pernot *et al.*, 2010 ▸). In an analogous manner to cryo-cooling in macromolecular crystallography (MX) (Garman, 1999 ▸), cryo-cooling samples down to 100 K for SAXS (cryoSAXS) has been reported to increase the dose tolerance of SAXS samples by at least two orders of magnitude (Meisburger *et al.*, 2013 ▸). Despite developments to improve the experimental apparatus for cryoSAXS (Hopkins *et al.*, 2015 ▸), it still requires specialized equipment and a certain level of technical expertise, which prevents it from currently being a commonly accessible technique (Jeffries *et al.*, 2015 ▸). Additives such as glycerol, ethyl­ene glycol, sucrose and sodium ascorbate can be added to the SAXS sample to increase the dose tolerance of the sample (Grishaev, 2012 ▸; Kuwamoto *et al.*, 2004 ▸). However, additives are also known to reduce the overall scattering signal (Jeffries *et al.*, 2015 ▸).

Application of the above radiation damage mitigation approaches are unable to completely circumvent its detrimental effects, in particular the change of the scattering profile throughout the experiment. It is necessary to determine whether any two scattering profiles are similar so that noise can be reduced by averaging over similar curves. The χ^2^ statistic, which describes the global goodness-of-fit of a model (Franke *et al.*, 2012 ▸), and the SAXS Merge method, which employs Gaussian process regression (Spill *et al.*, 2014 ▸), have previously been used to determine the similarity of scattering profiles. The CorMap test (Franke *et al.*, 2015 ▸) is the most recent method developed to assess the similarity of frames. Its advantage is that it does not use the errors on the experimental intensities, which are generally incorrectly estimated due to inaccurate propagation through the data processing pipeline.

For experiments by different researchers to be inter-comparable, the progression of radiation damage is most usefully tracked as a function of the dose absorbed by the sample. *RADDOSE-3D* is a free and open source software program used to calculate the time- and space-resolved three-dimensional distribution of the dose absorbed by a protein crystal in an MX experiment (Zeldin *et al.*, 2013*a* ▸). However, there is no equivalent software available for SAXS. Radiation damage studies in SAXS thus currently require the experimenters to correctly parameterize the experiment and manually calculate a single estimate of the dose within the sample (Hopkins & Thorne, 2016 ▸; Jeffries *et al.*, 2015 ▸; Meisburger *et al.*, 2013 ▸).

Here, extensions to *RADDOSE-3D* are presented, which enable the convenient calculation of doses for SAXS experiments, reducing the burden of manually performing the calculation. The three-dimensional geometry of the experiment is also taken into account. Furthermore, a new Python library has been developed to provide visual analysis of the results of scattering curve similarity tests from applying the CorMap test. Using this, it was possible to define a simple metric for significant radiation damage onset. These tools are used to compare the efficacy of eight different radioprotectant additives tested at various concentrations for their capability to increase the dose tolerance of glucose isomerase in a SAXS experiment. Additionally, the reduction in the scattered intensity signal is also analysed and the trade-off between dose tolerance and the signal reduction is briefly discussed.

## 2. Materials and methods   

### 2.1. Sample preparation   

Glucose isomerase (GI) from *Streptomyces rubiginosus* was chosen as the model protein for these experiments because it is a stable and soluble globular protein that has well defined SAXS behaviour, and it is sufficiently large to scatter well at modest concentrations (Kozak, 2005 ▸). It was purchased in tetrameric form (1552 residues, 172 kDa) from Hampton Research (GI mutant E186Q, now no longer commercially available) and was dissolved and dialysed for 24 h at 277 K against 100 m*M* HEPES and 10 m*M* MgCl_2_ buffer at pH 7.0. The final GI concentration, 1 mg ml^−1^, was used for all data collection runs and was determined using the known extinction coefficient, 45660 *M* ^−1^ cm^−1^ at 280 nm.

Eight soluble additives were tested over a range of concentrations for their radiation damage protection capabilities: di­thio­threitol (DTT), ethyl­ene glycol, glycerol, sodium ascorbate, sodium nitrate, sucrose, (2,2,6,6-tetra­methyl­piperidin-1-yl)oxyl (TEMPO) and trehalose.

The additives were added to the buffer solution (100 m*M* HEPES and 10 m*M* MgCl_2_ buffer at pH 7.0) without protein at four different concentrations: 10 m*M*, 5 m*M*, 2 m*M* and 1 m*M*, except for glycerol and ethyl­ene glycol, which were both prepared at 10% *v*/*v*, 5% *v*/*v*, 2% *v*/*v* and 1% *v*/*v* immediately prior to data collection.

These additives were also prepared to the same final concentration in the solution containing both the buffer and protein.

### 2.2. Data collection   

Data collection was performed at the ESRF BioSAXS beamline BM29 (Blanchet *et al.*, 2015 ▸; Brennich *et al.*, 2016 ▸; Materlik *et al.*, 2015 ▸; Pernot *et al.*, 2013 ▸). The photon energy used throughout was 12.5 keV and the photon flux was estimated from the beamline diode readings which were recorded for every frame using the conversion formula
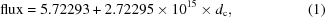
where 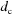 is the reading of the diode mounted within the backstop. The flux obtained using this formula was calibrated as described by Owen *et al.* (2009 ▸), here using an OSD1-0 photodiode purchased from Optoelectronics, which was a 500 µm-thick silicon diode with a 1 mm^2^ active area.

The data were recorded using a Pilatus 1M detector from Dectris. 15 µl of each sample was loaded into a 1.8 mm external diameter quartz capillary (1.7 mm internal diameter, thus the wall thickness is 50 µm) held at 20°C using the BioSAXS Sample Changer described by Round *et al.* (2015 ▸). For every additive, data were collected at each of the concentrations stated in §2.1, and each of these individual data collection runs was repeated three times (*i.e.* three datasets per radioprotectant per concentration). The exposure time for each frame was 1 s, and a total of 120 frames were collected for each repeat (*i.e.* 120 s total exposure for each dataset) with the sample kept static. After data collection on a sample, the capillary was washed with cleaning solution (2% Hellmanex, 10% ethanol and 88% distilled water), rinsed with distilled water and dried with dry air, a procedure also described by Round *et al.* (2015 ▸). For each radioprotecant concentration, a single dataset was collected with only the buffer (no protein) and the radioprotectant, so that a suitable buffer correction (subtraction) could be applied during data analysis. To obtain scattering curves on an absolute scale, a water calibration measurement was used.

### 2.3. Data processing   

Azimuthal integration of diffraction frames was performed as described in the corresponding section (§3.1) of Brennich *et al.* (2016 ▸). A custom script was written in Python to average the frames from the datasets collected with only the buffer with each radioprotectant added. These averaged frames were then subtracted from the frames collected with both the buffer and protein sample. Finally, the frames were cropped at both the lowest and highest scattering angles using the same Python script. The cropped scattering angles were the same for all datasets, and the choice of angles was determined by visual inspection to remove the regions with a higher level of noise.

### 2.4. Extending *RADDOSE-3D* for SAXS   

#### 2.4.1. Cylindrical sample geometry   

**Figure 1:**
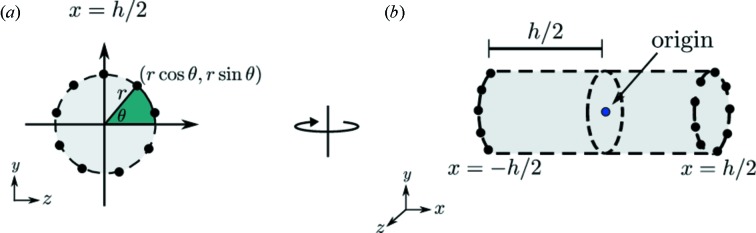
Implementation of the SAXS cylindrical sample geometry in *RADDOSE-3D* given a defined diameter, *d*, and height, *h*. (*a*) Evenly spaced points around a circle are generated given the radius, 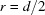, of the circular cross section. *RADDOSE-3D* defaults to 32 points. (*b*) In three dimensions the points represent the circles at each end of the cylinder at a distance of 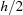 from the origin located at the centre of the cylinder.

In many SAXS experiments, liquid samples are contained in, or flowed through, a cylindrical capillary during the X-ray exposure, so it is necessary to model cylindrical sample shapes. To extend the capability of *RADDOSE-3D* to be used for the calculation of dose in SAXS experiments, a cylindrical shape geometry was implemented. It specifies the geometry of the sample alone, but not the capillary in which it is contained, and is graphically depicted in Fig. 1 ▸. (The effect of the capillary is dealt with separately in §2.4.3.) First, the points around a circle are generated using the diameter of the circular cross section. (*RADDOSE-3D* uses 32 points around the circle by default). The points are evenly spaced around the circle with 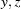 coordinates (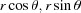). The angle (in radians) between any two consecutive points is 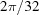. A cylinder can be defined by the circles at either end of the shape, so this is achieved using the final coordinate *x*. Depending at which end a particular point lies, it will have coordinates (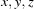) = (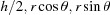) or (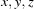) = (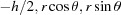). Note that *RADDOSE-3D* assumes that the origin of the system is located at the centre of the cylinder by default but that this can be changed by the user.

#### 2.4.2. Determining the sample composition   

The overall absorption coefficient of the sample, 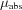, is calculated from the individual atomic absorption coefficients, 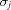, as
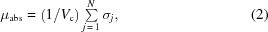
where 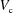 is the volume of the unit cell, *N* is the number of atoms in the unit cell and 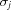 = 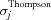 + 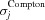 + 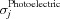 (Murray *et al.*, 2004 ▸). To determine the atomic composition of the sample, a volume of liquid is defined and its contents estimated, given its protein concentration and buffer composition.

First the molarity of the solution is calculated using the formula
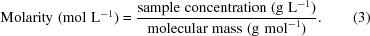
The sample concentration is provided in units of grams per litre (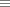 mg ml^−1^). The molecular mass of the molecule is calculated from other parameters provided. If the sequence file is given for the protein (the sample can also contain DNA and RNA), then the molecular mass can be determined by summing the molecular mass of each residue in the file. Otherwise, an average molecular weight is used for each residue (110.0 Da for protein residues, 339.5 Da for RNA nucleotides and 327.0 Da for DNA nucleotides).

The number of molecules in the volume can then be calculated by multiplying the molarity, volume and Avogadro’s number (*N* = 6.022 × 10^23^ mol^−1^), and the result is then rounded to the nearest integer.

#### 2.4.3. Beam attenuation due to the capillary   

In a typical MX experiment at 100 K, a crystal is exposed directly to the X-ray beam. In contrast, samples from SAXS experiments are held inside a quartz capillary. Thus the X-ray flux is attenuated due to the capillary, and account must be taken of this effect before calculating the dose absorbed by the sample. The transmission fraction of an X-ray beam due to a material with mass thickness *x* and density 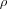 is given by
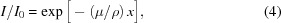
where *I* is the emergent intensity of the beam after penetrating the material, 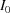 is the incident intensity and 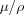 is defined as the mass attenuation coefficient (Hubbell & Seltzer, 1995 ▸). The mass thickness, *x*, is defined as the mass per unit area and is given by 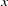 = 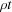 where *t* is the thickness of the material. The attenuation fraction caused by the capillary can hence be calculated as 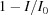.

The mass attenuation coefficients for each element are tabulated in the National Institute of Standards and Technology (NIST) tables. For mixtures, the total attenuation coefficient is given by
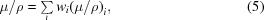
where 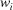 and 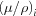 are the fraction by weight and the mass attenuation coefficient of the *i*th atomic constituent, respectively.

### 2.5. Dose calculation   

Doses were calculated using *RADDOSE-3D* (Zeldin *et al.*, 2013*a* ▸) and all doses referred to here are diffraction weighted dose (DWD) values (Zeldin *et al.*, 2013*b* ▸). The flux was calculated for each frame because the diode readings continuously changed between frames due to the decay of the electron current in the storage ring (see Fig. S1 of the supporting information). However, the overall change in diode current was only 0.54% during the course of a single run. Despite the small percentage change, this effect was still taken into account in the analysis.

A 100 µm-diameter circular aperture was scanned across the X-ray beam to obtain measurements of the beam profile. The readings were taken at 10 µm intervals with an OSD1-0 photodiode purchased from Optoelectronics. The scanning was performed six times with the collection of three horizontal and three vertical scans (Fig. S2 of the supporting information).

**Figure 2:**
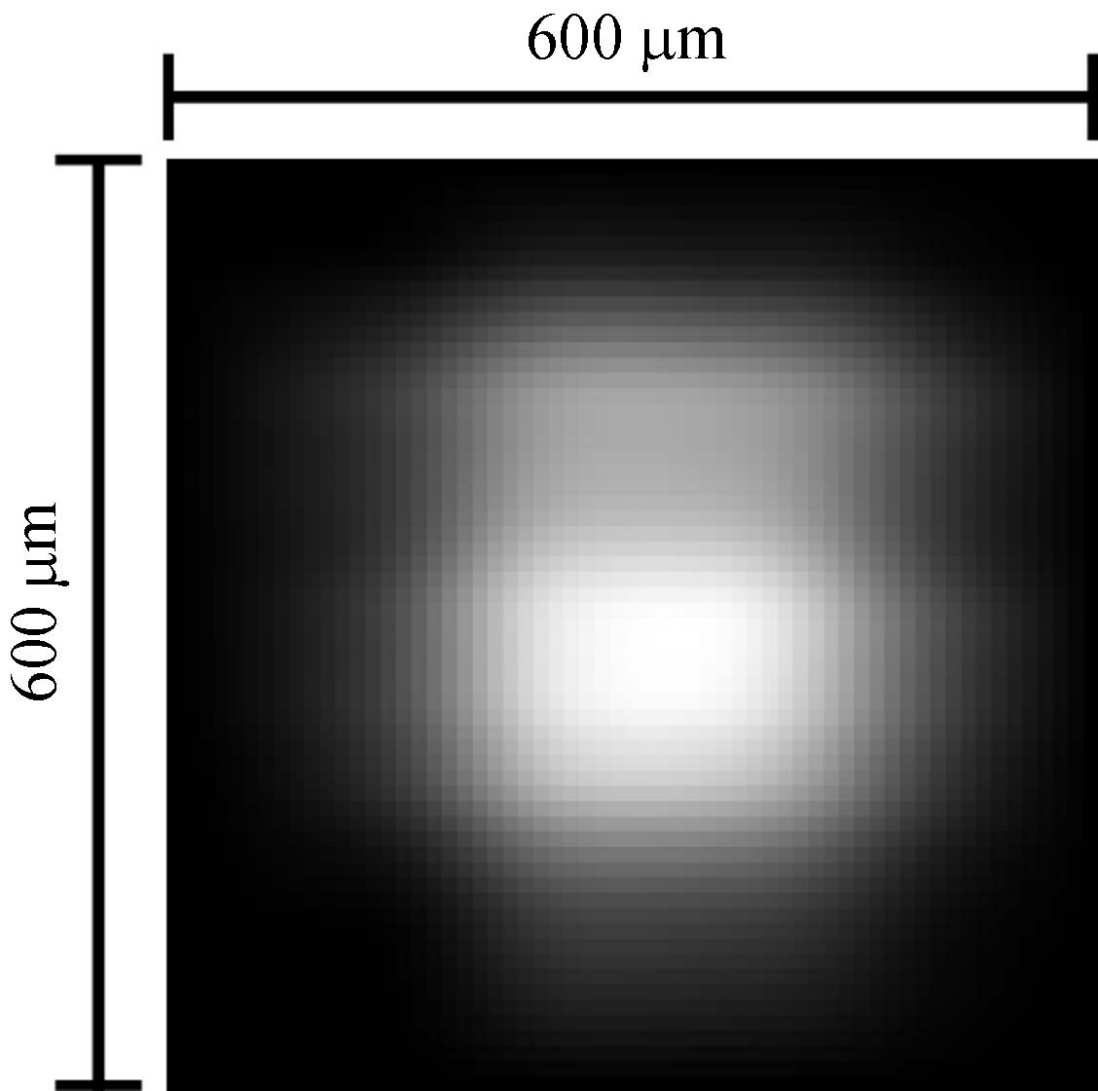
A two-dimensional reconstruction of the beam used in the experiment shown as a greyscale image. The intensity scales linearly between pixels.

To calculate a full two-dimensional beam profile from these aperture scans, a computational rectangular grid was constructed with the edges of the measurement positions used as the boundaries of the grid. The flux at and beyond the grid boundaries was assumed to be zero. The diode measurements from the vertical aperture scans were placed in their corresponding positions on the grid and interpolation between these values was performed using the ‘RectBivariateSpline’ function in the *SciPy* package in the Python programming language (Jones *et al.*, 2001 ▸). The same procedure was performed for the data in the horizontal direction. The interpolated two-dimensional beams were then averaged to obtain the final two-dimensional beam profile which was used in the *RADDOSE-3D* simulation (Fig. 2 ▸).

### 2.6. Radiation damage onset using the CorMap test   

The program *DATCMP*, distributed as part of the *ATSAS* suite of programs for processing SAXS data (Petoukhov *et al.*, 2012 ▸), was used to perform the one-dimensional scatter curve similarity analysis. *DATCMP* implements the CorMap test for assessing frame similarity (Franke *et al.*, 2015 ▸). This test performs a pairwise correlation between one-dimensional scattering curves derived from the diffraction frames, which involves taking the difference between the scattering curves. If the two curves are similar, and hence come from the same distribution, then the chance of observing a positive or negative value is 50%, which is the same chance as observing a head or tail when an unbiased coin is tossed. The Schilling distribution quantifies the likelihood of observing *C* number of heads/tails in a row, and this is extended to observing a given number of positive or negative values in the pairwise correlation map. The CorMap test calculates the probability, *p*, of observing the longest stretch of positive or negative values under the null hypothesis that the curves are similar. If *p* is above a given significance threshold, then the frames can be considered similar.

For the purposes of the current investigation, the first three frames of each experiment were compared in a pairwise manner using the CorMap test to ensure that they were similar (*p* value > 0.01). Then all subsequent frames were compared with frame 1. Radiation damage was assumed to have become significant at the point when three consecutive frames (in order to exclude outliers, for example bubbles or particles, passing through the beam) were found to be dissimilar as determined by the CorMap test at the *p* = 0.01 significance level. The dose absorbed in the sample for the first of the three consecutive dissimilar frames was then denoted the threshold dose, 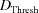.

### 2.7. Signal reduction   

As well as providing protection from radiation damage, the addition of a radioprotectant compound to the sample decreases the scattered intensity signal that constitutes the experimental data.

**Figure 3:**
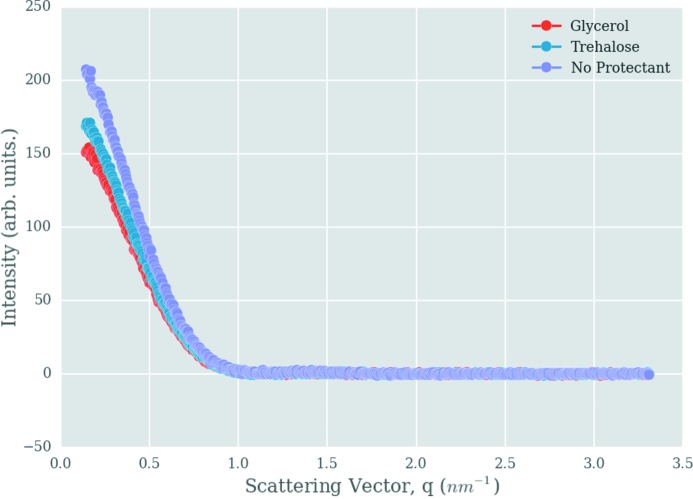
Scattered intensity signal reduction due to addition of radioprotectants. A reduction in the scattered intensity signal is seen when glycerol and trehalose are added as radioprotectants to the sample.

Fig. 3 ▸ shows the reduction in the scattered intensity signal when glycerol and trehalose are added as radioprotectants to the sample. To quantify this reduction, the ratio of the first 20 points (lowest *q* angles) of the samples containing the radioprotectant to the sample containing no radioprotectant was calculated for each of the eight compounds at each concentration. The mean of these values was then calculated and subtracted from 1 to give the average fractional reduction in the scattered signal. The lowest *q* values were chosen because that is where the signal reduction is most prominent.

## 3. Results   

### 3.1. CorMap visualization tools   

**Figure 4:**
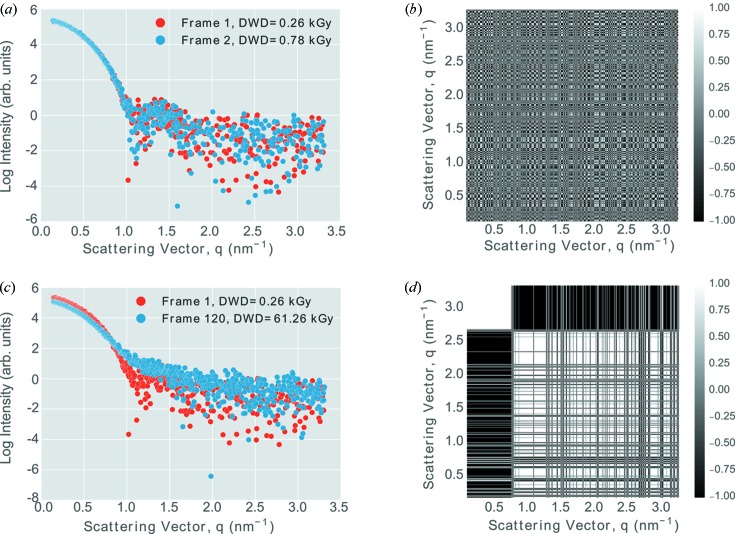
Similarity comparison with selected frames from the first experimental repeat for the GI protein and buffer but with no radioprotectant added. (*a*) One-dimensional scatter curves for frames 1 and 2. These two curves overlap well and are classed as similar. (*b*) Pairwise CorMap between frames 1 and 2. The ostensibly randomized lattice pattern suggests that the one-dimensional curves are similar. (*c*) One-dimensional scatter curves for frames 1 and 120. It is clear that these frames do not overlap. (*d*) Pairwise CorMap between frames 1 and 120. The dissimilarity between the two frames is represented by the large black and white regions.

A library for the visualization of the results of the CorMap test was developed, largely due to the fact that no open source alternative existed. One of the core visualizations is the correlation matrix between pairwise frames (Fig. 4 ▸). These correlation maps give an indication of the similarity of two frames. When two frames are similar, the correlation map resembles a randomized lattice (Figs. 4*a* and 4*b* ▸), whereas, when there are systematic differences between any two frames, there are large continuous regions of either black or white (Figs. 4*c* and 4*d* ▸).

**Figure 5:**
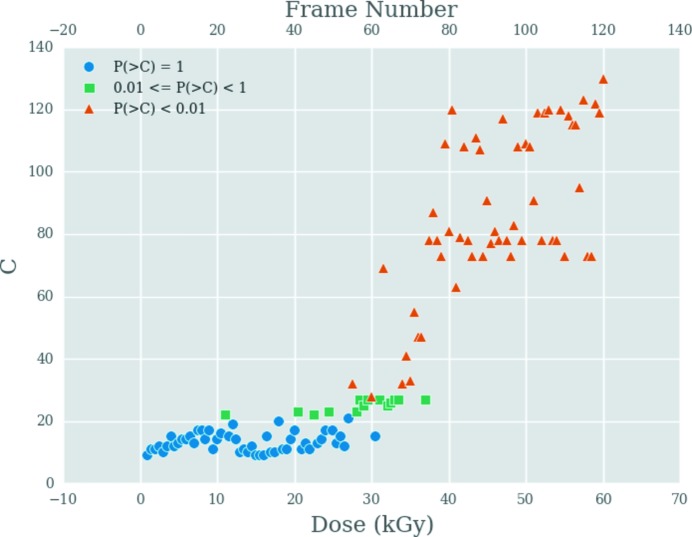
Longest observed black/white edge length in the correlation map, *C*, against the frame number and dose (kGy) for pairwise comparisons with frame 1. For similar frames to frame 1, the pairwise CorMaps resemble randomized lattices (Figs. 4*a* and 4*b* ▸) and hence *C* is fairly small. Therefore, the chance of observing a longer edge length than *C* is high [*P*(>*C*) = 1; blue circles]. As frames start becoming more dissimilar, the *C* values increase and the *P*(>*C*) values fall. These are represented by green squares. When frames are very dissimilar, *C* becomes very large (Figs. 4*c* and 4*d* ▸) and *P*(>*C*) < 0.01. The symbols representing the comparison with these frames are orange triangles.

Another visual representation developed for the current investigation which gives insight into the damage progression is the scatter plot showing the similarity of all frames with any chosen reference frame. Fig. 5 ▸ shows such a scatter plot where frames 2–120 are compared with frame 1. The variable *C* represents the length of the longest continuous patch of white or black in the corresponding pairwise correlation map. Each of the points is coloured by the *p* value of the CorMap test. If the *p* value is equal to 1, suggesting that the frames are similar, then the point is coloured blue, whereas if the *p* value is below a specified significance level (default level is 0.01) suggesting that the frames are different, then it is coloured orange. Points that have *p* values between a specified significance level and 1 are coloured green.

### 3.2. Concentration dependence of radioprotectant efficacy   


**Figure 6:**
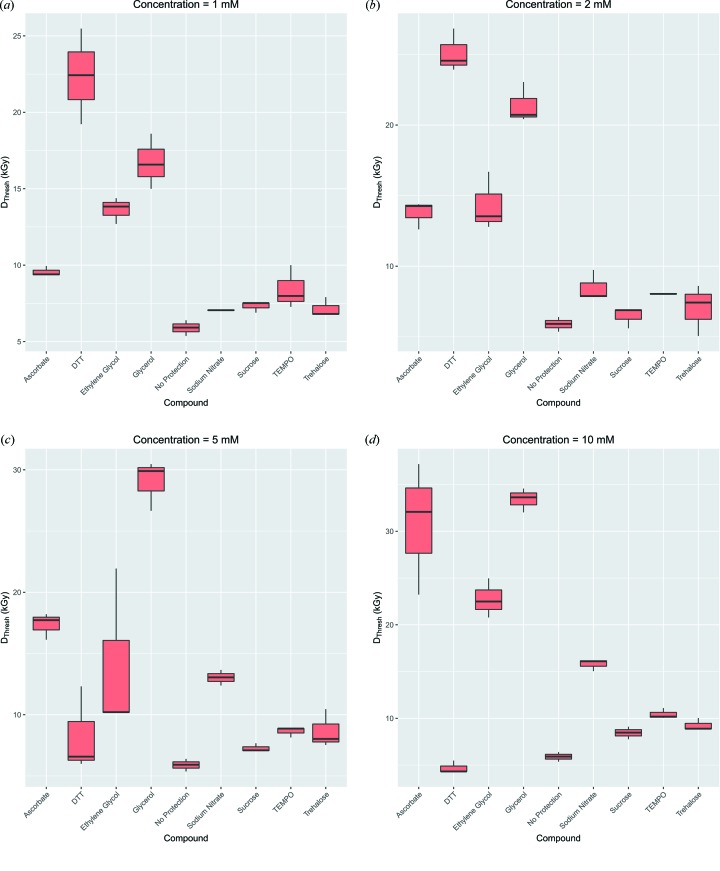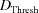 values as box plots for each of the three experimental repeats for each additive radioprotectant compound, including no additive at all. (*a*) 1 m*M*, (*b*) 2 m*M*, (*c*) 5 m*M*, (*d*) 10 m*M*.

**Figure 7:**
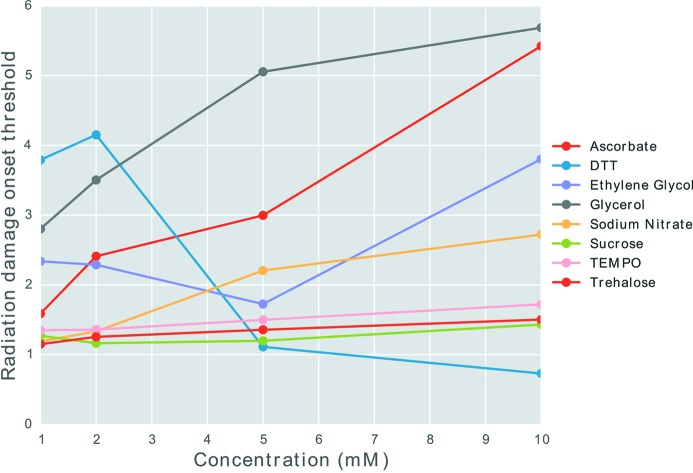
RDOT against concentration for the eight radioprotectants.

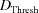
 values for each experimental run for each additive radioprotectant compound were calculated and are plotted in Fig. 6 ▸. These enabled the order of increased dose tolerance to be determined at each radioprotectant concentration; however, to quantify the improvement, a new metric called the radiation damage onset threshold (RDOT) was developed. This metric is defined as the ratio of the median 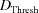 value with added radioprotectant to the median 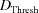 value for the same sample with no protection. Values below 1 correspond to a reduction in radiation tolerance whereas values above 1 show improved radiation tolerance. This metric was calculated for each compound at each concentration and the results are plotted in Fig. 7 ▸.

Significant concentration dependence can be observed for several of the radioprotectants. In particular, the efficacy of ascorbate, glycerol and sodium nitrate all exhibit a strong positive concentration dependence, *i.e.* the higher the concentration, the better the protection ability. However, DTT exhibits the opposite behaviour: at low concentrations it has the highest ratio, but this decreases at the higher concentrations. Sucrose, TEMPO and trehalose show a very small positive dependence but even at the highest concentration (10 m*M*) the RDOT is less than 2. This suggests that these radioprotectants are not very efficient at increasing the dose tolerance of the sample. The RDOT for ethyl­ene glycol decreases as the concentration increases from 1 to 5 m*M*, but then there is a large increase at 10 m*M*. Thus the protection ability of ethyl­ene glycol is not a simple monotonic function of the concentration.

The most effective radioprotectant varies depending on the concentration of the compound used in the sample. At low concentrations (1 and 2 m*M*) DTT is the most effective radioprotectant; however, at the higher concentrations (5 and 10 m*M*) glycerol becomes the most effective additive. Furthermore, the results also suggest that DTT is the least effective radioprotectant at the higher concentrations.

### 3.3. All pairwise frame comparisons should be made   

**Figure 8:**
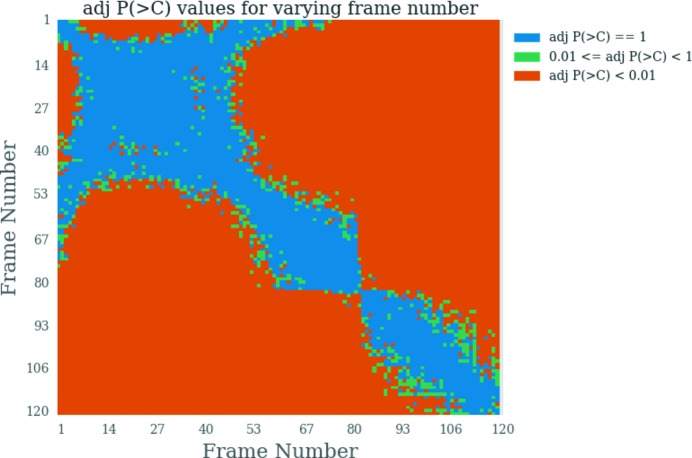
Heat map of all possible pairwise frame comparisons for the first repeat with 10 m*M* concentration DTT added to the GI sample. The *y*-axis represents the reference frame to which all other frames on the *x-*axis are compared. Blue: *P*(>*C*) = 1. Green: 0.01 ≤ *P*(>*C*) < 1. Orange: *P*(>*C*) < 0.01. The dose range is 0.29 kGy (frame 1) to 68.74 kGy (frame 120).

To understand why the efficacy of DTT changed so drastically as the concentration was increased, further investigation on frame combinations was undertaken. Rather than just taking pairwise frame comparisons with the first frame alone (which implicitly assumes that the first frame is undamaged), all possible pairwise comparisons were performed and plotted in a heat map for the data where DTT was added at 10 m*M* concentration (Fig. 8 ▸). The colour scheme is the same as that used for the scatter plot, namely blue, orange and green suggest, respectively, that the frames are similar, dissimilar or may be considered similar but that the *p* value is not equal to 1. The first row of the heat map gives the results of the CorMap test for all pairwise frame comparisons with the first frame. It can be seen that a region of orange begins from frame 8 (DWD = 4.32 kGy), demonstrating why 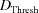 was so low for 10 m*M* DTT. The large blue square region in the top left of Fig. 8 ▸ suggests that these frames are all similar, and so much more useful data can be obtained if frames 7 (DWD = 3.74 kGy) to 57 (DWD = 32.50 kGy) are used for merging. This means that assessing all pairwise comparisons, as opposed to pairwise comparisons with the first frame only, may result in better quality data and improve the consistency of the radiation damage analysis.

### 3.4. Reduction of scattered intensity signal   

**Figure 9:**
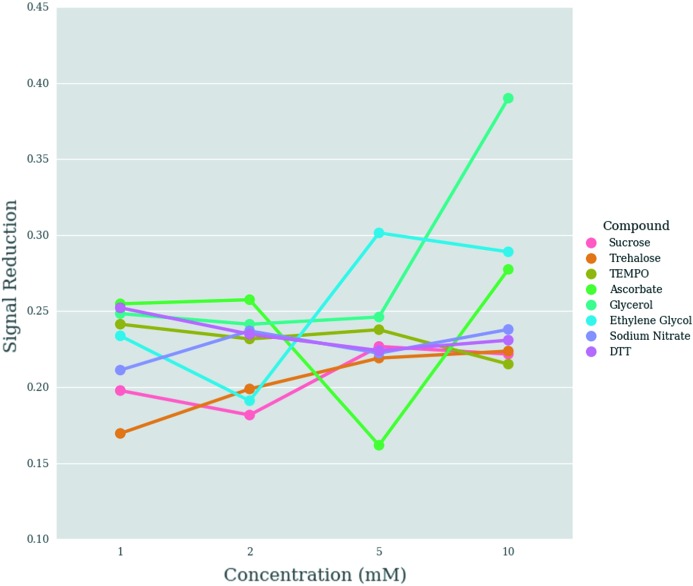
Scattered intensity signal reduction for each radioprotectant (see text for method of calculation).

Fig. 9 ▸ shows the reduction in the scattered intensity signal for each radioprotectant. The majority of the data show a reduction between 16 and 26% (for *q* values from 0.14 to 0.23 nm^−1^). The compounds for which the signal reduction exceeds 26% are glycerol, sodium ascorbate and ethyl­ene glycol, all of which were the best radiation tolerant compounds at concentrations of 5 and 10 m*M*, according to the RDOT metric. Notably, at 5 m*M* concentration, sodium ascorbate appears to reduce the signal the least, despite being the second most radiation tolerant additive of those used in our experiment. However, counterintuitively, at the lower concentrations (1 and 2 m*M*), it decreases the intensity signal more than any of the other compounds. This suggests that there may be a non-linear relationship between signal reduction, radiation tolerance and concentration. DTT, sodium nitrate and TEMPO exhibit a slightly negative correlation between the signal reduction and the concentration, *i.e.* as the concentration increases, the reduction in scattered intensity signal decreases.

## 4. Discussion   

The work presented here describes how extensions to *RADDOSE-3D* to simulate SAXS experiments, along with additional analysis of results from the CorMap test, can be used to perform quantitative radiation damage analysis of SAXS experiments. In particular, the three major additions to *RADDOSE-3D* were:

(i) Implementation of a cylindrical sample geometry.

(ii) Determination of the sample composition given a mg ml^−1^ protein concentration.

(iii) Attenuation of the X-ray beam due to a surrounding capillary.

In isolation, the CorMap test performs pairwise comparisons of frames. This means that an individual frame can be detected as being dissimilar to another frame regardless of the similarity assessment of frames immediately before and after the frame in question. This dissimilarity of an individual frame relative to its neighbour therefore may not indicate a true systematic change in the molecules of the sample; rather, it may just be an outlier. Thus, a more robust indicator for radiation damage in this work was defined as the point whereby three consecutive frames were assessed as being dissimilar to the first frame.

The additions described above allowed the convenient visualization of the results from the original CorMap test, and thus enabled the comparison of the efficacy of eight different additive radioprotectant compounds for their ability to improve the dose tolerance of a protein sample (glucose isomerase, GI). It was established that some radioprotectant compounds exhibit a stronger concentration dependence than others in their ability to increase the dose tolerance of GI. Explicitly, at the lower concentrations, DTT was the most effective radioprotectant whereas, at the higher concentrations, glycerol was the most effective, resulting in more than a fivefold improvement in dose tolerance at 10 m*M* concentration.

Furthermore, the visualization library developed for this work was able to highlight why DTT performed poorly, and show that performing all pairwise comparisons could result in more consistent frame-merging methods.

Despite the extensions described above, the SAXS data collection model in *RADDOSE-3D* makes some implicit assumptions. For instance, with regard to the capillary, the atomic composition is assumed to be uniform throughout, and the thickness to be constant around the entire sample volume. The advantage of these assumptions is that the attenuation by the capillary only needs to be calculated once, regardless of any movement or rotation of the capillary. This is valid for a cylindrical capillary since the thickness penetrated by the X-ray beam is the same regardless of any rotations or translations.

Additionally, the sample itself is assumed to be static, moving as a rigid body when rotated or translated, and also to be completely filling the capillary. This greatly reduces the computational cost when compared with the possibility of modelling more realistic fluid dynamics. The assumption that the capillary volume is completely filled is generally valid since this is usually the case during an experiment. The static assumption, on the other hand, is not always appropriate, especially when the sample is flowed through the capillary. Hopkins & Thorne (2016 ▸) calculated that for typical experimental parameters the velocity profile is expected to exhibit the quadratic Poiseuille flow profile, which arises from the axial symmetry and no-slip boundary assumptions (the velocity at the centre of the tube moves the fastest while the velocity at the boundary is equal to zero provided the capillary is also stationary). Hopkins & Thorne also calculated that the residence times of the sample in the beam were too short for any appreciable radial diffusive mixing, so that the flow profile results in radius-dependent residence times in the X-ray beam. Therefore, the static assumption made in *RADDOSE-3D* will give misleading dose values if calculated for experiments where the sample is flowed through the beam position.

Diffusive turnover is another phenomenon that will affect the dose calculation. Molecules have the ability to diffuse into and out of the illuminated volume, with the additional complexity that a non-uniform beam profile will cause differential diffusion across the beam due to higher sample heating at the peak of the beam profile. In the current work, no account was taken for molecular diffusion. However, beam sizes in SAXS experiments are typically quite large and exposure times are not long enough for the diffusive turnover to cause a significant effect. For example, Jeffries *et al.* (2015 ▸) used a 500 µm × 250 µm sized beam with a maximum exposure time of 141 s per sample while irradiating at 283 K (Jeffries *et al.*, 2015 ▸).

An important consideration regarding the concentration dependence of the efficacy of the additives is that they can alter the preferred environments of the sample. For example, it is known that DTT reduces disulfide bonds and undergoes oxidation. Glycerol at higher concentrations, on the other hand, is known to increase the noise and hence reduce the observable signal obtained from the sample (Jeffries *et al.*, 2015 ▸). In our study the reduction in the scattered intensity signal was examined for the different radioprotectants. Generally, the best performing radioprotectants, in terms of increasing the radiation tolerance of the sample, also reduced the scattered signal most. However, this order is not necessarily consistent, which suggests that the relationship is likely to be non-linear. Therefore, striking the optimal balance between increasing radiation tolerance and the level of signal reduction will still require some level of experience and input from the experimenter.

The definition of the signal reduction used in this study only considered the differences of the scattered intensity curve at the lowest *q* values where the difference was most prominent. However, the shape of the one-dimensional scattering curve can vary dramatically for different protein samples. Therefore, a metric for assessing the reduction that faithfully accounts for the difference across the entire *q* range may be more desirable.

Another important factor to consider is that the efficacy of radioprotectants may alter for different protein samples. This will likely be due to the various interaction processes that will occur for different macromolecules. For example, radioprotectants that performed best in this study could be involved in the prevention of the GI tetramer oligomerization breakdown. This process may not be as important for other samples, and hence the order of efficacy found in this study may not be applicable to a different sample.

During the data processing stage, the data were cropped by visual inspection to remove noisy sections. This is a subjective choice and therefore reduces the reproducibility of this work. To overcome this problem, quantitative criteria could be applied to find the optimum cut-off values for the data. Investigation of these possible criteria should be considered for further investigation of this method.

The 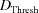 values in our study range between 2.37 and 51.24 kGy. These differ significantly from the various threshold values in other types of diffraction experiments: room-temperature MX ∼150–500 kGy (Roedig *et al.*, 2015 ▸; Southworth-Davies *et al.*, 2007 ▸), cryo-crystallography ∼30 MGy (Owen *et al.*, 2006 ▸) and cryo-SAXS >3.7 MGy for µL samples and between 100–300 kGy for nL samples (Meisburger *et al.*, 2013 ▸). These differences are likely to be attributable to the differences in the experimental factors, *e.g.* temperature, sample type *etc*. However, there is still significant variation in the critical doses calculated for other room-temperature SAXS experiments [∼400 Gy (Kuwamoto *et al.*, 2004 ▸) and 284–7700 Gy (Jeffries *et al.*)] when compared with the 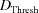 values calculated in our study. Some of the variation can be attributed to the different protein samples and concentrations used in the experiments for the studies. However, the most likely cause of the apparent discrepancy is the various definitions of the critical dose. Hopkins & Thorne (2016 ▸) show that applying the critical dose definition from Jeffries *et al.* (2015 ▸) to their own GI data results in a critical dose of ∼66000 kGy, whereas the range in the Jeffries *et al.* (2015 ▸) study for GI was 5964–7056 Gy. Furthermore, they also state that ‘the molecular weight shows a significant (13%) change after just ∼75 kGy’. Hence the critical dose value is highly dependent on the definition chosen to calculate it. Additionally, differences can also be expected due to the fact that the critical dose estimates from the previous studies are based upon (pseudo) radius of gyration values, whereas in our study the 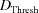 values are determined by analysis of frame similarity.

Although these issues should be taken into account for any given experiment to ensure optimum and successful data collection, the ability to predict the dose expected in a BioSAXS experiment and to rationally choose a suitable additive to improve the starting conditions offers considerable benefits for data quality and efficiency of BioSAXS experiments. The methods and tools presented in this paper can be used in a complementary manner to other metrics created for assessing the quality of SAXS data (Grant *et al.*, 2015 ▸; Hopkins & Thorne, 2016 ▸).

The CorMap analysis visualization source code is freely available on Github: https://github.com/GarmanGroup/CorMapAnalysis.

## Supplementary Material

Additional text and figures supporting the information in the main text. DOI: 10.1107/S1600577516015083/xh5050sup1.pdf
