# Supplementary material for: Development of tools to automate quantitative analysis of radiation damage in SAXS experiments
Source: J Synchrotron Radiat. 2017 Jan 1;24(Pt 1):63–72. doi: 10.1107/S1600577516015083 (PMC5182020; doi:10.1107/S1600577516015083)
Supplement: Supplementary file 1 [file s-24-00063-sup1.pdf]

JOURNAL OF SYNCHROTRON RADIATION

Volume 23 (2016)

Supporting information for article:

Development of tools to automate quantitative analysis of radiation damage in SAXS experiments

Jonathan C. Brooks-Bartlett, Rebecca A. Batters, Charles S. Bury, Edward D. Lowe, Helen Mary Ginn, Adam Round and Elspeth F. Garman

Figure S1 Diode readings for each frame during the first experimental repeat for the GI sample with no radioprotectant added (total exposure time is 120 seconds). It can be clearly seen that the diode readings decrease throughout the experiment. The total change is about 0.54%.

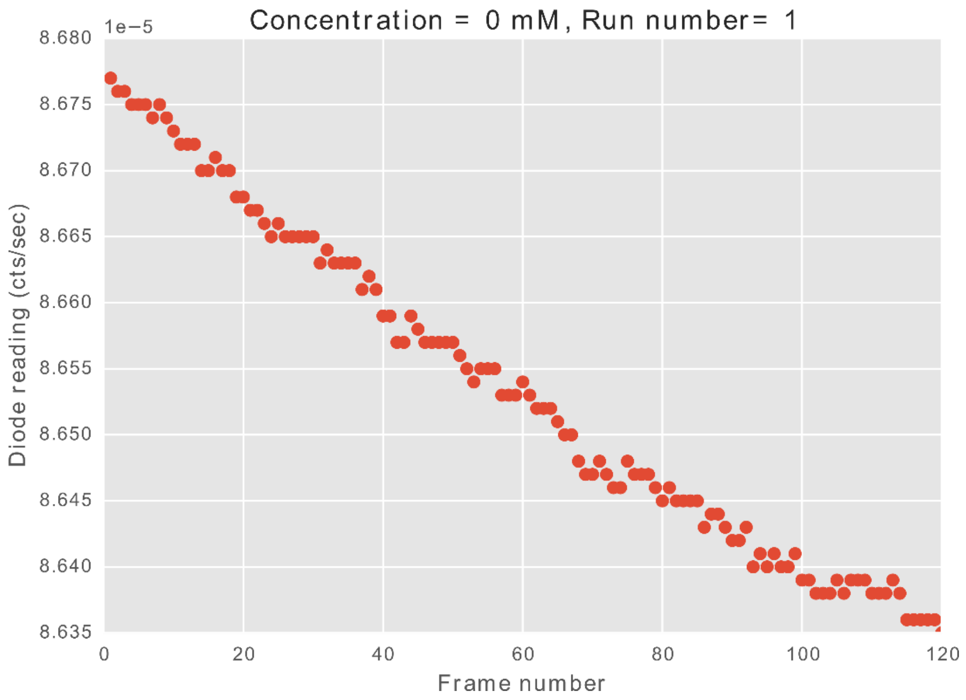

Figure S2 Diode readings from aperture scans across the beam collected at beamline BM29, ESRF, displayed in Figure 2 of the main text. (a) Vertical scan through the centre of the beam. (b) Horizontal scan through the centre of the beam. 4 other scans (2 vertical, 2 horizontal at 10 µm intervals) were also taken at other locations across the beam.

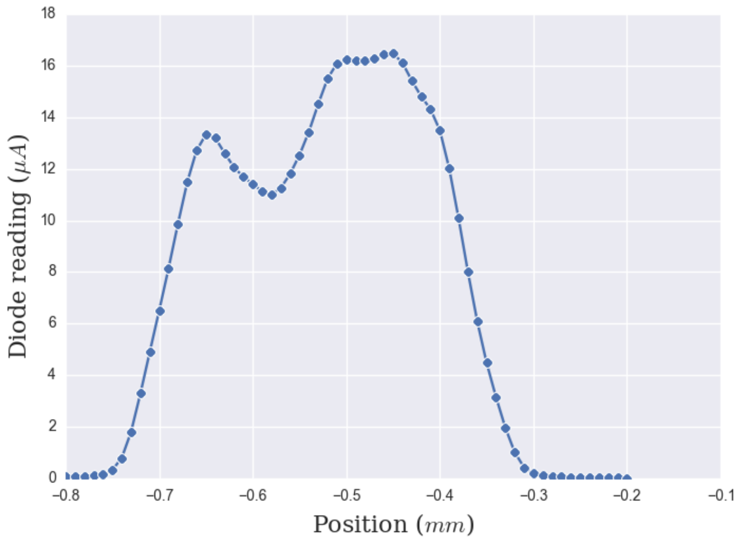

(a)

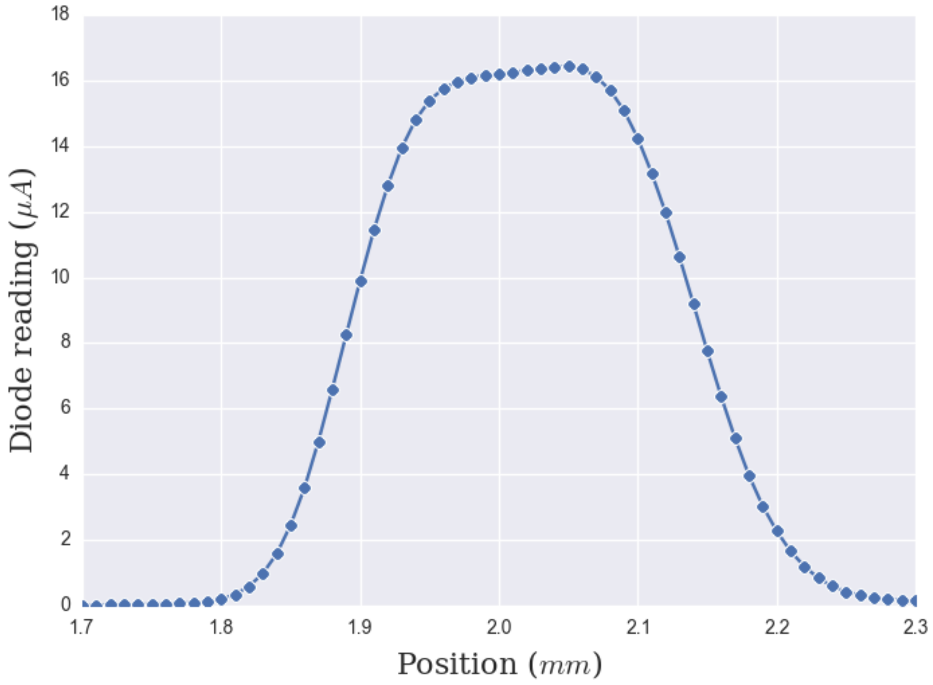

(b)

Figures S3 and S4: Defining the radiation damage onset

For the work presented in the main text, radiation damage onset was assumed to have become significant at the point when three consecutive frames were found to be dissimilar, as determined by the CorMap test at the p = 0.01 significance level. To ensure that these criteria were reasonable and fairly stable, it was necessary to consider the different parameter values, namely to test various numbers of consecutive dissimilar frames, denoted m, and a range of significance levels, denoted p. Figure S3 shows the resulting D_{Thresh} values with $p = 0.01$ and various values of m.

Figure S3 shows that for most radioprotectant compounds, the spread of the apparent radiation damage onset is generally larger for $m = 1$ than for $m = 3, 5, 7, 10$. For $m = 3, 5, 7, 10$ the values corresponding to the onset of significant radiation damage are practically identical (except for DTT). Thus for the radiation damage analysis $m = 3$ was used.

Figure S4 shows the D_{Thresh} values with $m = 3$ and $p = 0.01, 0.05$ and 0.1 for all the radioprotectant data.

It can be seen that the median values are very similar for the various p values. Given this fact and that $p = 0.01$ is the recommended (and sufficiently strict) threshold (Franke *et al.*, 2015), $p = 0.01$ was chosen as the value used for all radioprotectant compounds in the radiation damage analysis. The advantage of using the $p = 0.01$ threshold is that frames have to be very dissimilar before the calculated p value falls below the threshold. This means that it is less likely that frames are discarded when they actually are similar (in statistical speak this means there is less chance of a type I error).

Figure S3 D_{Thresh} values for each radiation protectant against m, the number of consecutive dissimilar frames, for the 8 radioprotectants tested.

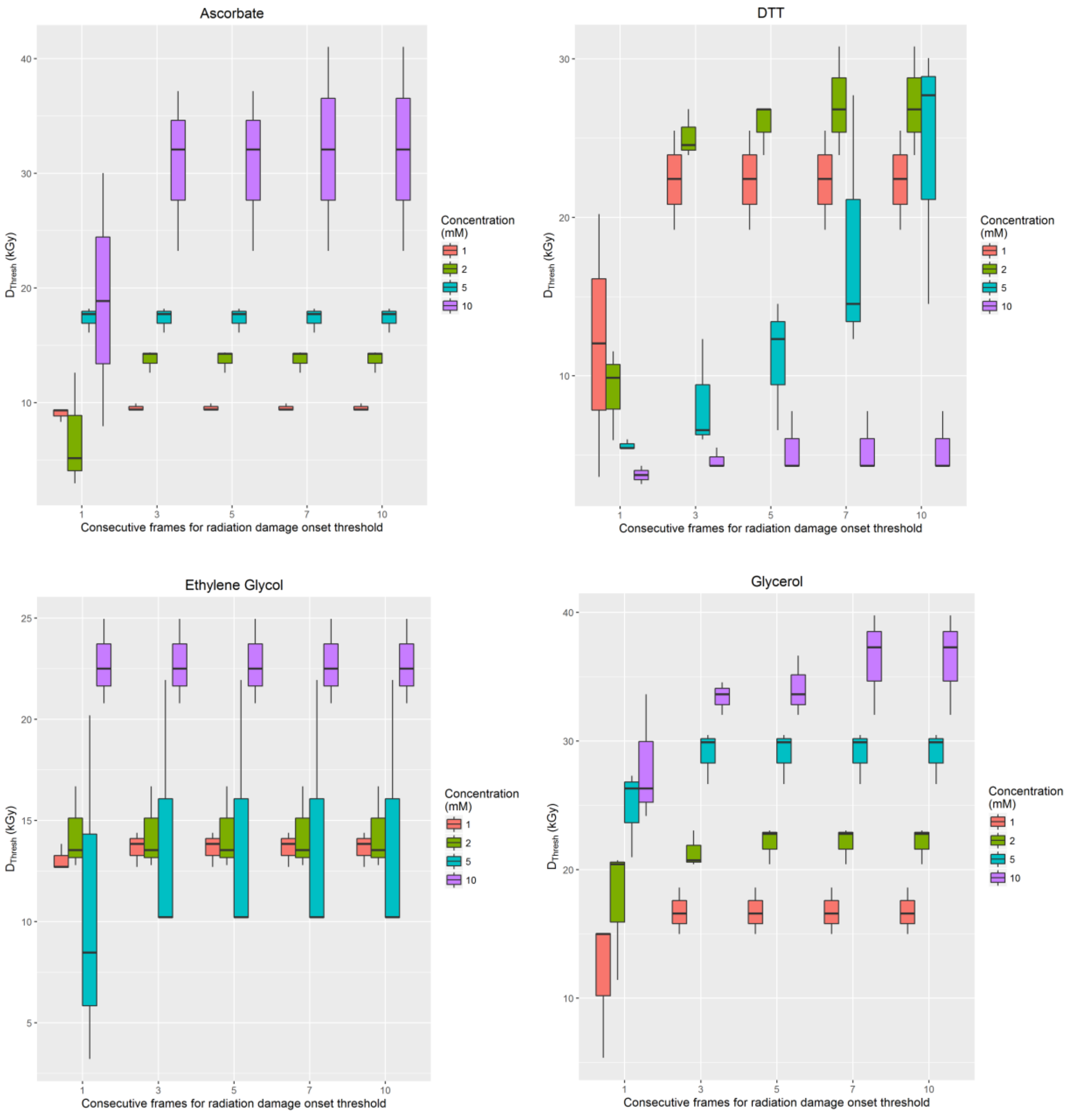

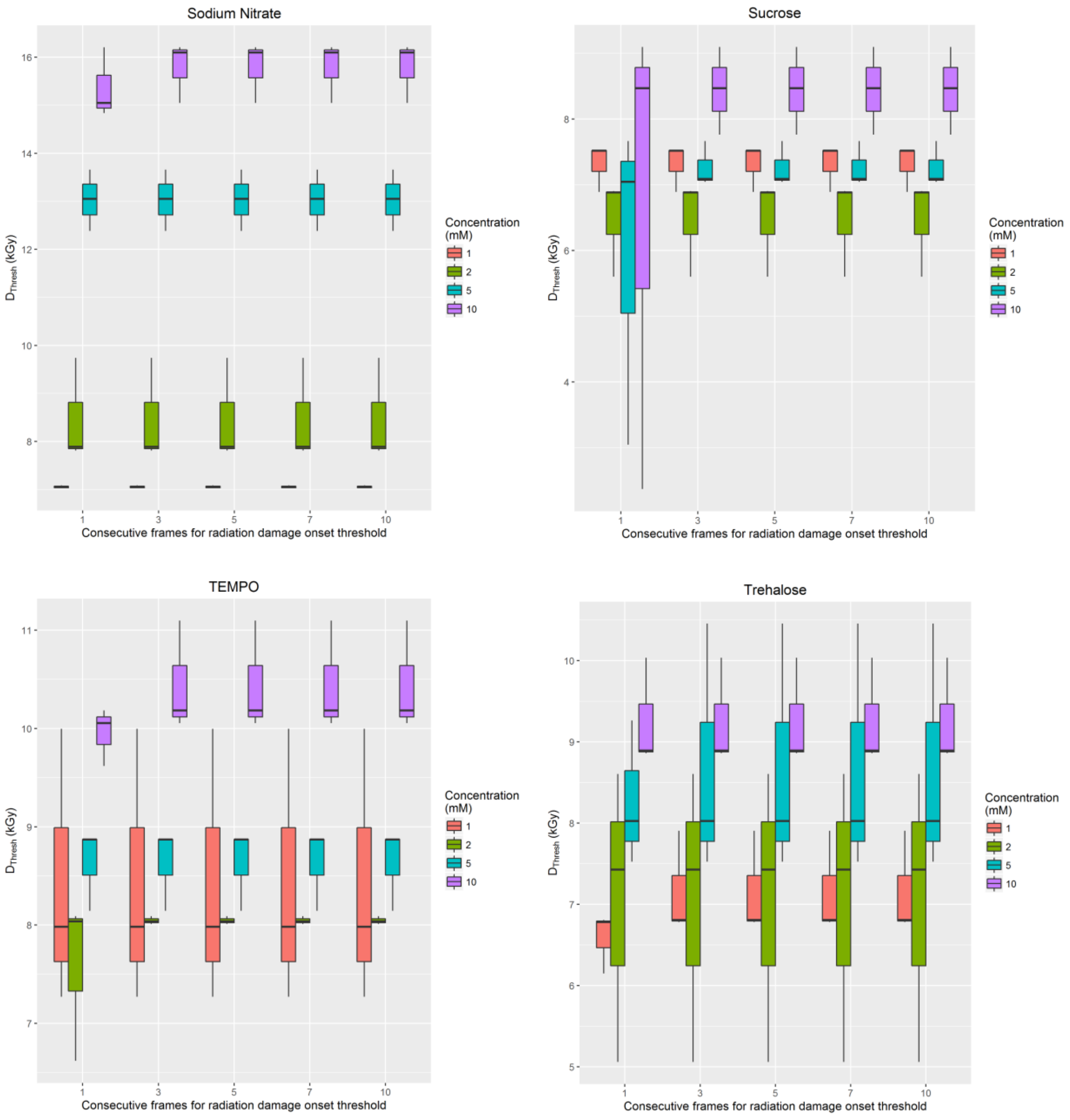
Sodium Nitrate
DThresh (kGy)
Consecutive frames for radiation damage onset threshold
Concentration (mM)
1
2
5
10
Sucrose
DThresh (kGy)
Consecutive frames for radiation damage onset threshold
Concentration (mM)
1
2
5
10
TEMPO
DThresh (kGy)
Consecutive frames for radiation damage onset threshold
Concentration (mM)
1
2
5
10
Trehalose
DThresh (kGy)
Consecutive frames for radiation damage onset threshold
Concentration (mM)
1
2
5
10

Figure S4 D_{Thresh} values for each radiation protectant against p value for the 8 radioprotectants tested.

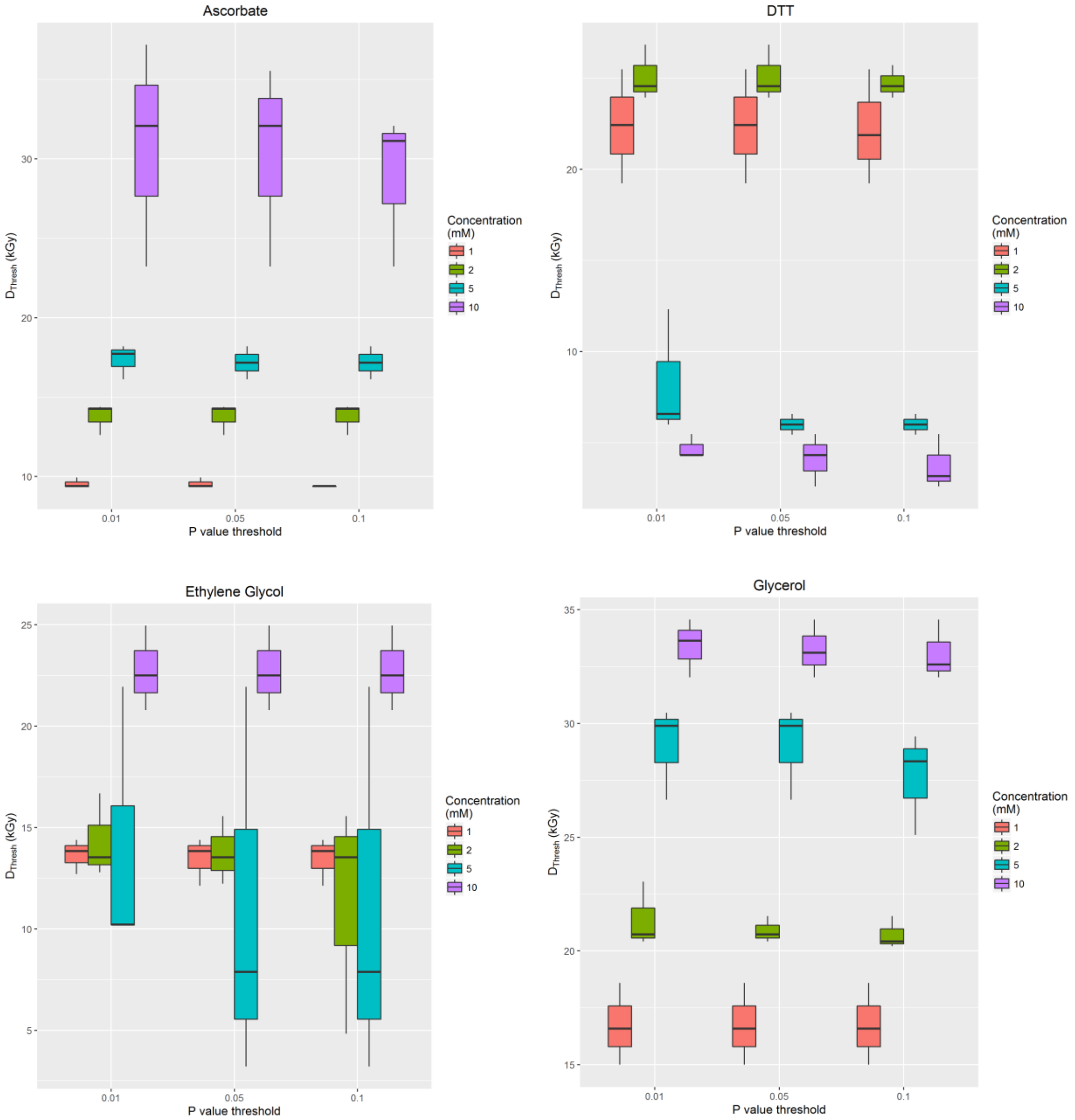

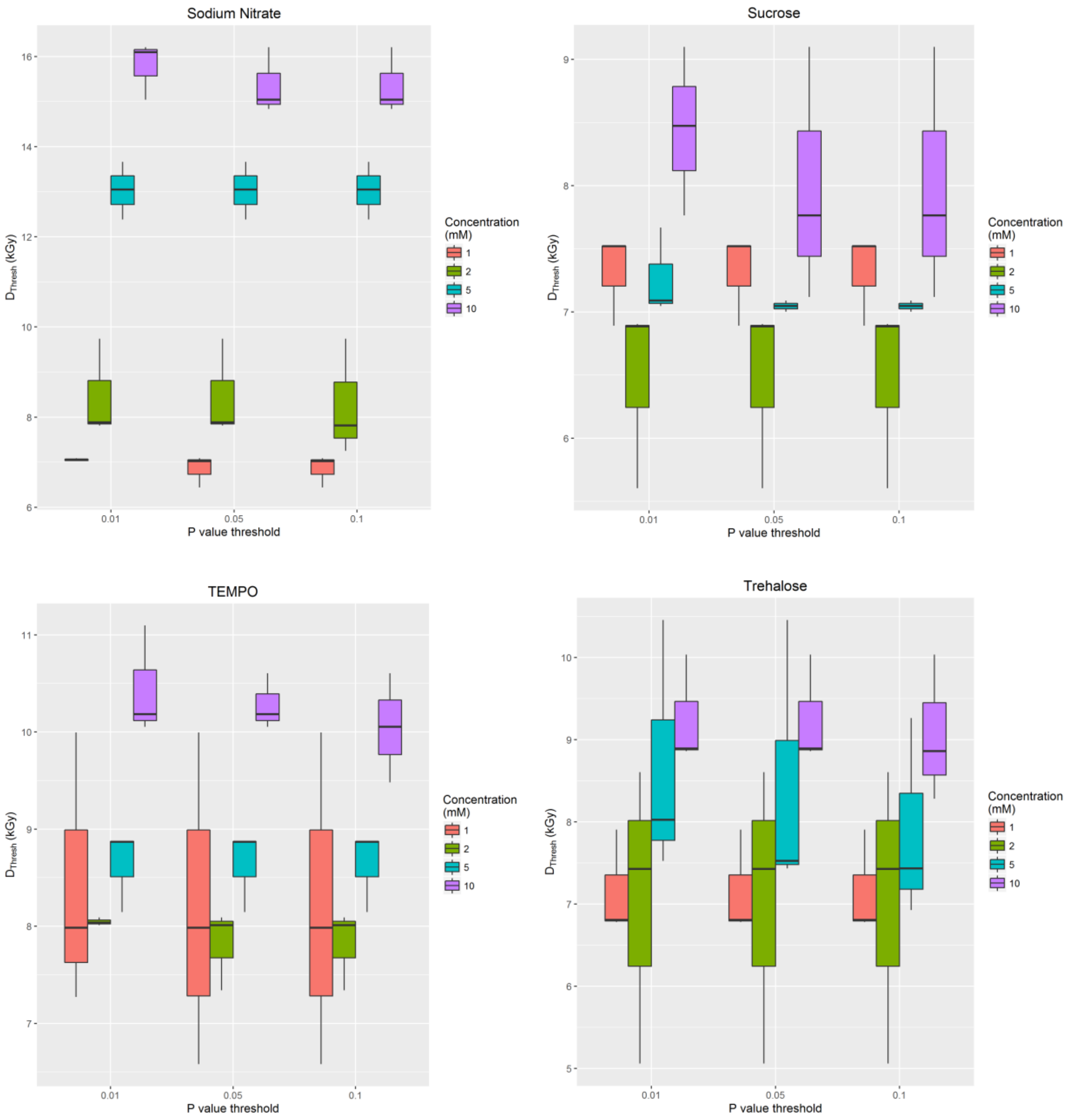
Sodium Nitrate
Sucrose
TEMPO
Trehalose
D_{Thresh} (kGy)
P value threshold
0.01
0.05
0.1
Concentration (mM)
1
2
5
10

References

Franke, D., Jeffries, C. M. & Svergun, D. I. (2015). *Nat. Methods*, **12**, 419-422.
